# Subtle Cr isotope signals track the variably anoxic Cryogenian interglacial period with voluminous manganese accumulation and decrease in biodiversity

**DOI:** 10.1038/s41598-019-51495-0

**Published:** 2019-10-21

**Authors:** Lingang Xu, Anja B. Frank, Bernd Lehmann, Jianming Zhu, Jingwen Mao, Yongze Ju, Robert Frei

**Affiliations:** 1 State Key Laboratory of Geological Processes and Mineral Resources, China University of Geoscience, 100083 Beijing, China; 2 Department of Geosciences and Natural Resource Management, University of Copenhagen, 1350 Copenhagen, Denmark; 3 Mineral Resources Unit, Technical University of Clausthal, 38678 Clausthal-Zellerfeld, Germany; 4 China Aero Geophysical Survey & Remote Sensing Center for Land and Resources, 100083 Beijing, China

**Keywords:** Element cycles, Mineralogy

## Abstract

Earth’s atmosphere experienced a step of profound oxygenation during the Neoproterozoic era, accompanied by diversification of animals. However, during the Cryogenian period (720–635 million years ago) Earth experienced its most severe glaciations which likely impacted marine ecosystems and multicellular life in the oceans. In particular, large volumes of Mn and Fe accumulated during the interglacial intervals of the Cryogenian glaciations, indicating large anoxic marine metal reservoirs. Here we present chromium isotope-, rare earth element-, and redox-sensitive trace element data of sedimentary rocks from the interglacial Datangpo Formation deposited between the Sturtian and Marinoan glaciations in South China, in an attempt to investigate the oxidation state of the oceans and atmosphere. Both the Cr isotope and trace element data indicate mainly anoxic water conditions with cryptic oxic surface water incursions after the Sturtian glaciation. Glacial-fed manganese precipitated as manganese carbonate in anoxic basins, and the non-fractionated δ^53^Cr record of −0.10 ± 0.06‰ identifies anoxic conditions with a cryptic component of slightly fractionated Cr isotope composition in manganese ore, in line with distinctly fractionated Mo isotope composition. Both the manganese carbonate ore and the black shales exhibit very low redox-sensitive element concentrations. Our study demonstrates that the oxygenation of the seawater, and inferably of the atmosphere, at the beginning of the Cryogenian interglacial interval was much subdued. The post-glacial rebound then allowed the Ediacaran biological diversity.

## Introduction

**Figure 1:**
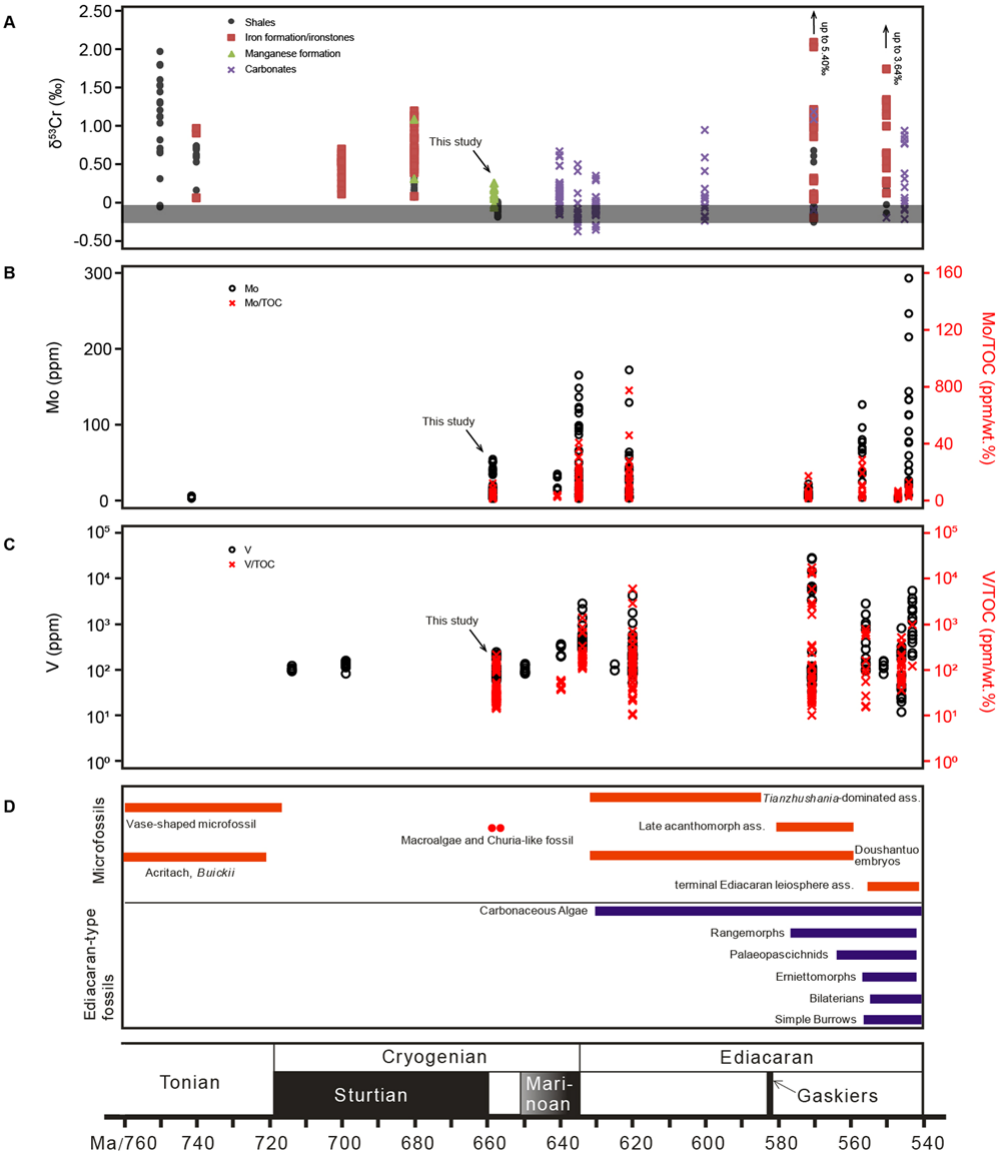
Compilation of sedimentary δ^53^Cr and redox sensitive element data in the context of biological evolution across the 750–540 Ma interval. (**A**) Chromium isotope data of shales, iron formations, manganese formations, and carbonates. The horizontal gray field denotes the δ^53^Cr values of igneous silicate Earth (ref.^47^). (**B**) Temporal trends in Mo enrichments (circles) and Mo/TOC (crosses) in black shales. (**C**) Temporal trends in V enrichments (circles) and V/TOC (crosses) in black shale. (**D**) Diversified appearance of microfossils in pre-Cryogenian marine sediments, followed by decrease of biological diversification during the Cryogenian period, which is then characterized by the “Ediacaran biological diversity” (ref.^48^). The Cryogenian interglacial period of the Datangpo Formation in South China records macroalgae and Churia-like fossils at the base (red solid dots), followed by rare fossils at the upper part (ref.^27^). Data compilation, stratigraphic and chronological details are provided in the Supplementary Information. The δ^53^Cr data indicate that the ocean experienced low oxygenation in the aftermath of the Sturtian glaciation and followed by anoxic conditions till the Ediacaran, in line with evidence from Mo and V records, which document slight enrichment only at the base of the Datangpo Formation, followed by consistently low abundances of Mo and V, and their ratios with TOC.

Oxygen concentrations in the atmosphere and in the surface ocean waters have played a significant role in controlling the evolution of life on Earth. For example, the emergence of eukaryotes and complex multicellular organisms is consistent with two oxygenation events that occurred ~2.4 billion years ago and during the later Neoproterozoic era, respectively^1^. The Cryogenian period was a time of turbulent environmental change, dominated by two significantly large and widespread glaciation events – the Sturtian- and Marinoan glaciations. Biodiversity between the two glaciations was comparatively depressed, with only a few microfossil assemblages documented (ref.^2^, Fig. 1). Although there was an overall increasing oxygen concentration in the atmosphere and oceans during the Neoproterozoic era that was in path with the evolution of macroscopic multicellular life^2–4^, the oxidation seems not to have taken place in a unidirectional and stepwise increasing manner^5,6^. The sedimentary sequences formed during the Sturtian-Marinoan interglacial interval either document drawdown of biodiversity^2,7^ or the rise of algae^8^, exhibiting instabilities in the level of biodiversity, which potentially links to fluctuations in atmosphere and ocean oxygenation.

Manganese (Mn) and iron (Fe) are geochemically similar elements; their accumulation in the oceans shows a strong relationship with the redox state of the waters, and ultimately with the level of oxygen in Earth’s atmosphere^9,10^. The Neoproterozoic is an era during which Mn deposition has peaked, e.g., co-deposition of Mn-oxides with BIF interbeds, such as in Namibia and Brazil, and pure Mn-carbonate (rhodochrosite; MnCO_3_) accumulations in India and South China^11^. In the Nanhua basin, South China, giant Mn deposits are associated with interglacial sediments deposited after the Sturtian glaciation. Although a hydrothermal origin of Mn has been a popular scenario to explain the formation of these manganese deposits^12,13^, continental sources and anoxic marine redox conditions (refs^14,15^) may provide an alternative explanation for their formation.

Chromium (Cr) isotopes have been promoted as a novel proxy for characterizing paleoenvironmental changes in ancient oceans, offering a link to past changes in atmospheric and marine oxygenation levels, due to the highly sensitive response of Cr in the surface environment during oxidative weathering processes. Chromium has two stable oxidation states in nature – the soluble Cr(VI) anions (CrO_4_^2−^, or HCrO_4_^−^) and the insoluble Cr(III) compounds. Most Cr in Earth’s crust is in rock-forming minerals in its reduced and insoluble state Cr(III). Under oxidative weathering surface conditions, the insoluble Cr(III) can be oxidized to Cr(VI). The oxidative weathering process results in the preferential release of isotopically heavier Cr and its mobilization into solution as Cr(VI) compounds, which then are readily delivered to the ocean via riverine transport. In oxic seawater, Cr has a residence time of 3,000 to 40,000 years^16–18^. Changes in the marine oxidation state can lead to significant isotope fractionation as Cr(VI) reduction preferentially affects lighter Cr isotopes, leading to enrichment of isotopically heavy Cr in the remaining unreacted Cr(VI) pool^19–21^.

The stripping of isotopically heavy Cr from seawater into sediments, for example by OM reductive capturing, can potentially lead to distinctively fractionated δ^53^Cr values in the authigenic fraction of reduced marine sediments (i.e., black shale), compared to the igneous silicate Earth pool^22^. A positively fractionated authigenic Cr fraction therefore potentially indicates the existence of isotopically heavy surface water, and indirectly, the existence of an oxidized atmosphere that enabled the mobilization of isotopically heavy Cr form the landmasses. The absence of such positively fractionated signals in reduced sediments, in contrast, would support the prevalence of rather low oxygenated atmospheric conditions^23–26^.

Here we report chromium isotope data of the Datangpo Formation in the Nanhua Basin, South China, comprising an interglacial marine sedimentary succession deposited between the Sturtian and Marinoan glaciations. In combination with REE and other redox-sensitive element data, we attempt to characterize the ocean redox conditions in the Nanhua Basin, in which a large volume of manganese accumulated at the beginning of the Cryogenian interglacial period. Our data on a total of 38 bulk rock samples plus 12 leachates provide a more direct assessment of the oxygenation of the basin waters in the transition between the Sturtian and Marinoan glaciations, and provide potential links to the decrease of biodiversity and to the deposition of giant manganese mineralization during this time interval.

## Geological Setting and Samples

**Figure 2:**
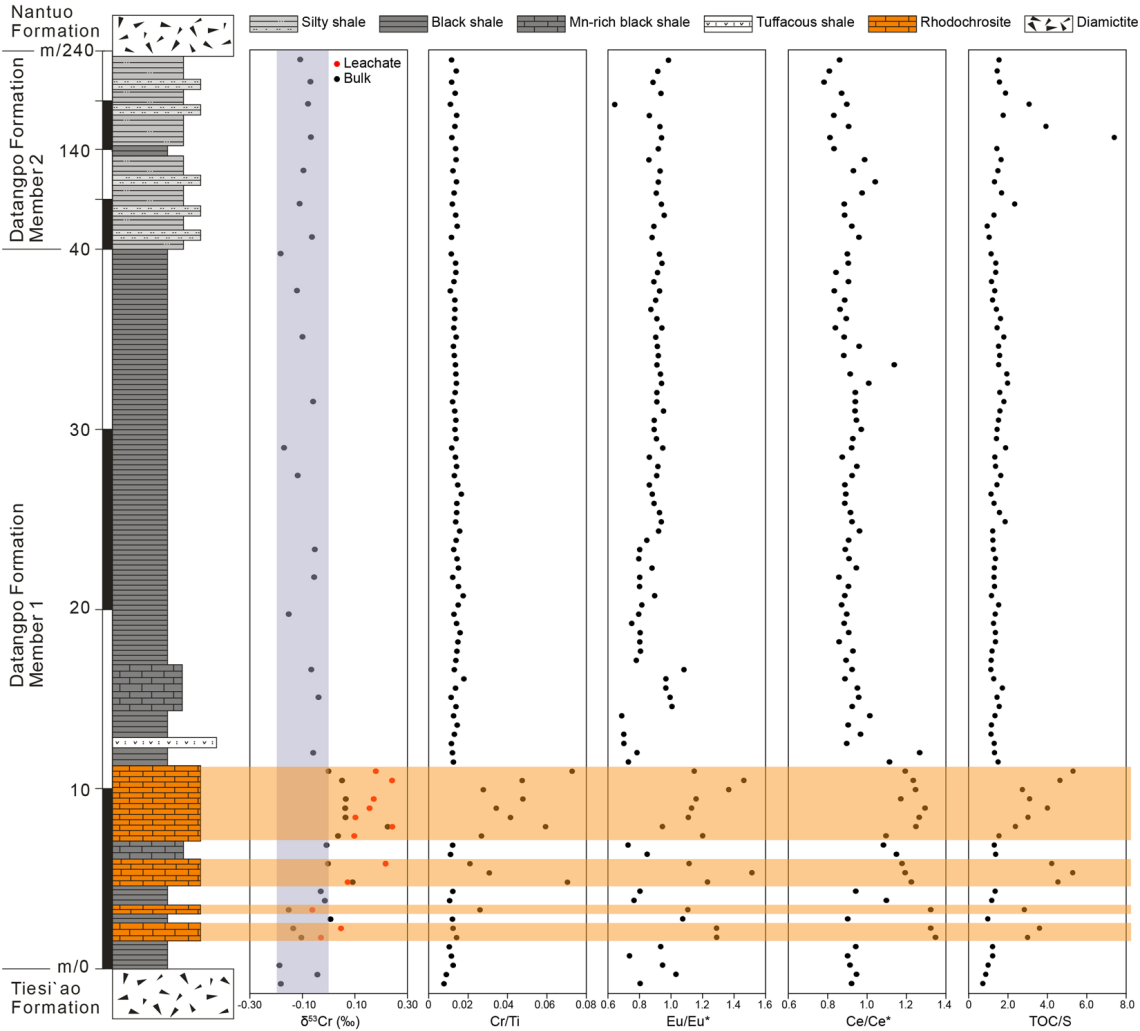
Geochemical signals for rhodochrosite and black shale samples from the Datangpo Formation, Nanhua Basin. The gray vertical bar represents the crustal average value (ref.^47^). The light orange horizontal bars denote rhodochrosite layers.

The Datangpo Formation in the Nanhua Basin comprises integrated interglacial marine platform-margin deposits between the Sturtian and Marinoan glaciation (Supplementary Information). It is characterized by macroalgae and Churia-like microfossils in the lower part but by rare fossil occurrences in the upper part of the formation^27^. Samples studied herein of the Datangpo Formation are from a drill core in the Songtao area, 400 km northeast of Guiyang city, the capital of Guizhou province, China. Zircons of tuff layers in the lower and upper horizons of the Datangpo Formation yielded U-Pb ages of 663 ± 4 Ma and 655 ± 4 Ma^28,29^, implying a sedimentation period that lasted a maximum of 16 Ma. The Datangpo Formation is underlain by the Tiesi’ao Formation and overlain by the Nantuo Formation, two diamictite formations that correspond to the global Sturtian and Marinoan glaciations, respectively (Supplementary Information). The interglacial Datangpo Formation contains two lithological members – Mb.1 consisting of 40-m-thick black shales, and Mb.2 consisting of 190-m-thick silty shales that overlie the Mb.1 (Fig. 2 and Supplementary Information). The basal part of the Mb.1 is the host of giant manganese ore deposits. The Mn ore is dominated by stratiform manganese carbonate (mainly rhodochrosite, MnCO_3_) layers of variable thickness (0.5–4 m), and is interbedded with laminated carbonaceous shales. Rhodochrosite occurs in oolitic texture and cemented by manganocalcite (Supplementary Fig. 3).

## Cr Isotope Data and Ocean Redox Reconstruction

**Figure 3:**
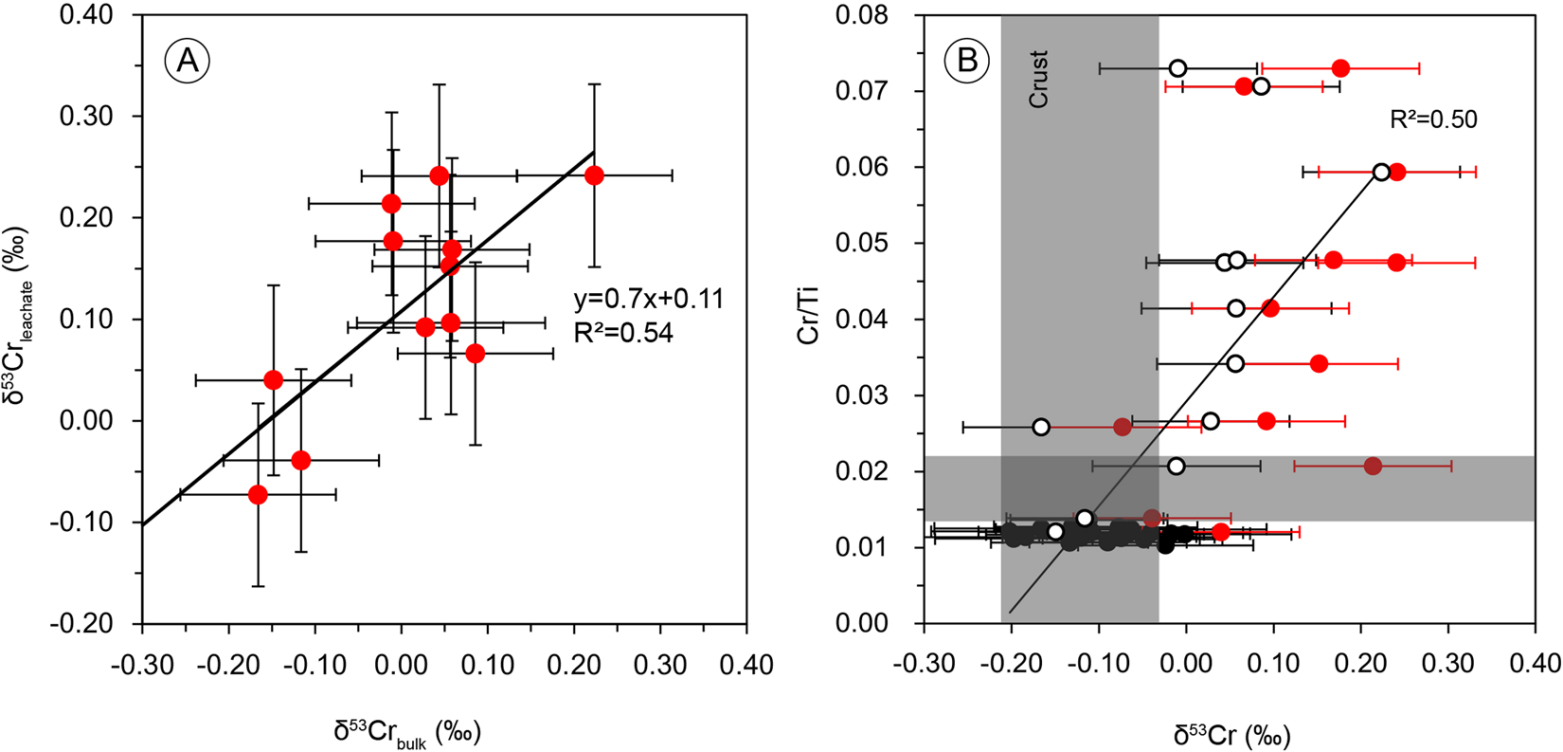
(**A**) Correlation between bulk and leachate δ^53^Cr data for rhodochrosite samples with a positive shift by a mean of 0.11‰ for the leachates, indicating that the leaching method is efficient to enhance the authigenic Cr isotope fractionation signature. (**B**) Cr/Ti ratios and δ^53^Cr values with internal reproducibility (2σ). The Cr/Ti ratios have a positive correlation with the bulk δ^53^Cr values (R^2^ = 0.50). The gray fields denote the crustal average values. δ^53^Cr data of rhodochrosite samples are displayed for bulk rock (black circles) and leachates (red solid dots). The black solid dots represent the unmineralized black shales.

The bulk chromium isotope signature of a sediment sample results as the weighed sum of contributions from authigenic and detrital components. In shale samples particularly, authigenic Cr isotope signatures are often obscured by signals from detrital materials. As our interest lies in the characterization of authigenic components, and in order to minimize contributions to the Cr signatures stemming from detrital components, we also conducted some leachate analyses, designed to preferentially attack the Mn-carbonate fraction of rhodochrosite ore samples. The black shale samples have a δ^53^Cr isotope mean of −0.10 ± 0.06‰ (n = 14), identical with silty shale of −0.10 ± 0.02‰ (n = 7), and within the range of unfractionated silicate bulk Earth of −0.12 ±0.10‰ (Fig. 2). The Cr isotope data on rhodochrosite ore gave a δ^53^Cr isotope mean value of 0.01 ± 0.10‰ (n = 12), which is slightly heavier than the black shales. The leachate fractions on the same rhodochrosite samples gave 0.11 ± 0.10‰ and the data show a proportional relationship between the bulk and the leachate δ^53^Cr signatures, with systematically positively fractionated values in leachates by a mean factor of about 0.1‰ (Fig. 3) compared to bulk samples.

Our Cr isotope data suggest overall anoxic marine conditions for the time of deposition of the manganese ore. There is, however, a cryptic signal of Cr isotope fractionation in the samples, because there is a systematic positive shift in Cr isotope composition from black shales to rhodochrosite ore of about 0.1‰, plus a positive shift of the same order of magnitude from the bulk rhodochrosite ore to the leachate fractions. However, these subtle differences in δ^53^Cr data allow no clear identification of oxic conditions and limit the redox conditions to close to the aqueous Cr^4+^/Cr^6+^ equilibrium which is close to the Mn^4+^/Mn^2+^ boundary^30^. The δ^53^Cr values identify an oxidation state of atmosphere and ocean much lower than for Phanerozoic and modern black shales (ref.^31^). However, the interpretation of a cryptic weakly oxic signal is probably right, given the available Mo isotope data on the Datangpo Formation, which gave a mean δ^98^Mo value of +1.06 ± 0.06‰ (SD) for the Mn-bearing black shales compared to the overlying black shales with average δ^98^Mo value of +0.65 ± 0.31‰ (ref.^32^). These data can be interpreted as indicating mildly oxic seawater which delivered Mo to a euxinic environment during manganese ore formation, followed by a larger anoxic seawater reservoir which deposited less fractionated Mo in the overlying black shales.

We also observe a weakly defined positive correlation between the δ^53^Cr values and Cr/Ti ratios in the manganese ore samples (R^2^ = 0.50, Fig. 3). This relationship is consistent with mixing of authigenic Cr of positively fractionated Cr signature and a detrital component with the composition of the igneous Earth inventory. Elevated Cr/Ti ratios in black shales have been interpreted to derive from co-precipitation and scavenging of particle reactive Cr(III) phases and/or sorption and sequestration of dissolved Cr(VI) onto OM and subsequent shuttling into the sediment^17^. The unmineralized black shale samples studied herein are characterized by even lower Cr/Ti ratios than the present-day upper continental crust^22^, indicating a very limited or no authigenic Cr proportion in these samples, while the Mn ore samples show a weak but distinct authigenic Cr component. In addition, we observe a negative correlation between Cr and Eu/Eu* (R^2^ = −0.50), which also suggests binary mixing between components from authigenic manganese carbonates and black shales (Supplementary Fig. 4).

The unfractionated Cr isotope signatures of black shale samples from interlayers and from above the rhodochrosite layers could a priori be interpreted by an overwhelming co-sedimentation of isotopically unfractionated Cr(III) associated with detrital components. Low concentrations of redox sensitive elements (e.g., Mo and V) and their low ratios with TOC of marine black shales have been observed throughout Earth history before the Ediacaran, and interpreted as the result of low oxygen concentration in ocean and atmosphere^33,34^. The generally low concentrations of redox sensitive elements of sediments of the Datangpo Formation indicate the prevalence of anoxic conditions, also during the sedimentation of black shales studied herein (Supplementary Fig. 5). The systematically low TOC/S ratios provide independent evidence for such anoxic conditions during the precipitation of the Datangpo black shales (Fig. 2). The global sulfur cycle is intimately linked with marine redox conditions, with low total sulfur content in marine oxic sediments in which oxidative biodegradation of organic matter is likely to happen^35^. Therefore, the relatively higher TOC/S ratios recorded in the manganese ore of the Datangpo Formation may point to a weakly increase of oxygen concentration in the water column during Mn mineralization, although we cannot rule out lithological and diagenetic effects imposed on the manganese ore samples to have caused this. Nevertheless, the basal black shales show slightly elevated but still low Mo and V compositions and ratios with TOC compared to Ediacaran black shale (Fig. 1B,C), indicating only weakly oxic conditions at the beginning of the interglacial interval. The black shale - manganese ore interlayers could be interpreted as an expression of weakly oxidative pulses and compositional changes in the water column after the Sturtian glaciation. Mn ore was deposited during periods of seafloor oxygen incursions, which enabled the precipitation of manganese carbonates. The massive black shale depositional interval overlying this perturbed sequence then shows the return to more stagnant and pervasive anoxic water column chemistry lasting until the onset of the Ediacaran.

## Manganese Formation

The compiled geochemical data of the manganese ore and associated black shales show a strong negative correlation of MnO content with Al_2_O_3_ and SiO_2_ (Supplementary Fig. 6A,B). Aluminum in marine sediments is overwhelmingly derived from continental weathering of silicates and of detrital nature. A negative correlation between MnO and Al_2_O_3_ (and likewise with SiO_2_) contents is therefore evidence that manganese in the sediments and the Mn-ores are not associated with continental detrital components. The manganese ore samples are mixtures of detrital and biogenic/authigenic components. Consequently, also rare earth element signatures of manganese ores do reflect these mixtures. Our compiled data show positive correlations between MnO and Eu/Eu*, and between MnO and Ce/Ce* (Supplementary Fig. 6C,D). Positive Eu anomalies have been regarded as one of the characteristic features of modern and ancient chemical sediments of submarine hydrothermal exhalative origin^36^. For example, the presence of high-temperature hydrothermal vent fluids in the depositional environment of most Archean and Proterozoic BIFs is indicated by positive Eu anomalies in their REE patterns^37^. The rhodochrosite samples studied herein from the Nanhua Basin show positive Eu/Eu* anomalies between 1.11–1.52, with one exception of a sample exhibiting a Eu/Eu value of 0.94. Such positive Eu/Eu* values could be interpreted to show a subaqueous hydrothermal imprint on the respective water column from which these carbonates precipitated^12,13^. However, there is no evidence for the former presence of vent outlets and associated alteration features in the sediments, or hydrothermal minerals in the sedimentary record. An alternative explanation for the positive Eu/Eu* anomalies characterizing these carbonates is mixing of glacial-fed continental water with seawater. Such a process is commonly characterized by enrichment of middle REE and positive Eu/Eu* anomalies in the precipitates, as observed for modern glacial-fed rivers^38^. Although the authigenic signature of manganese carbonates studied herein is largely obscured by detrital components, we still observe weak positive enrichments of middle REE (Supplementary Fig. 7) in the respective patterns. We see these features as supporting a glacial-fed river water - seawater mixing model to explain the formation of the Mn-rich carbonates during the aftermath of the Sturtian glaciation. Glacial-fed river water input to the ocean may have been pervasive globally in response to an increase in global temperatures during the interglacial interval.

Independent evidence comes from Ce anomalies. Ce exists in either a trivalent or a tetravalent form, and in oxygenated water soluble Ce^3+^ tends to be adsorbed to Fe and/or Mn (oxyhydr)oxide particles on which oxidation to the highly insoluble Ce^4+^ is catalyzed, eventually resulting in positive Ce anomalies in sediments, leaving an overlying water column characterized by negative Ce anomalies^39^. Positive Ce anomalies are found in modern hydrogenetic Fe-Mn crusts that precipitated under oxidized seawater conditions^40^. Archaean and Early Proterozoic marine sediments systematically lack negative Ce anomalies, implying that the ambient seawater was not sufficiently oxidizing to form Ce^4+^ (ref.^41^). Therefore, the positive Ce anomalies in the rhodochrosite samples (values between 1.09–1.35) are indicative of an oxidized upper water column during Mn deposition, a scenario which is in line with our model. We note that most black shale samples studied herein exhibit slight negative Ce anomalies (Supplementary Fig. 6D), also suggesting oxidative pulses at the beginning of the aftermath of the Sturtian glaciation.

## Implications for Co-Evolution of Ocean Anoxia, Bioproductivity, and Mn Precipitation

Macroalgae and abundant Churia-like fossils have been described from within the manganese ore layers in the lower part of the Datangpo Formation in the Nanhua Basin^27^. The appearance of these fossils is in line with the oxidative surface seawater conditions during the formation of manganese ore revealed by our Cr isotope and trace element geochemical data, and previous Mo isotope data (ref.^32^). The black shales lack such fossils^27^, in accordance with pervasive anoxia in the depositional environment which prevented the thriving of oxygen respiring organisms. The decrease of biodiversity and ocean productivity during the Cryogenian interglacial period is not unique to South China records, but other world-wide sedimentary sequences covering this interval have revealed similar results^7,42^. This suggests that the chemical and biological evolution during the Cryogenian interglacial period were probably intrinsically linked on a global scale.

The manganese ore in the Nanhua Basin contains pyrite concretions with anomalously positive S isotope composition (up to 65‰, ref.^13^), suggesting a restricted marine basin situation. Manganese carbonate was either precipitated near the water–sediment interface under dysoxic conditions (ref.^43^) or during early diagenetic carbonation^12^. On the basis of our data, we consider a seawater redox-control model for the formation of the giant manganese ore deposits in the Nanhua Basin. At the beginning of the aftermath of the Sturtian glaciation, greenhouse conditions may have been pervasive globally, and potentially led to enhanced glacial-fed river water input into the oceans, ultimately allowing for significant accumulation and concentration of Mn^2+^ in seawater. The periodically weakly oxic conditions in the aftermath of the Sturtian glaciation allowed near-surface organic matter production and decay under anoxic conditions with CO_2_ from methanogenesis, which precipitated Mn^2+^ on the seafloor or below as manganese carbonate. Light carbon isotope composition (δ^13^C down to −10‰, ref.^13^) of the manganese carbonate ores suggests a carbon source from oxidation of organic material by methanogenesis processes. Although Mn^2+^ is soluble^44^, precipitation of manganese carbonate is still possible as reported in many ancient analogues^9,11^. In cases where sediment pore waters are Ca-rich, mixed Ca-Mn carbonates commonly form, as observed in the Keogas, Molango, Urucum, and Hotazel manganese deposits^45^. The oolitic texture of rhodochrosite suggests that precipitation of manganese carbonate occurred in shallow seawater, within the wave base and a shoaling water column. Diagenetic processes unlikely affected the Cr isotope system in the sediments, as Cr was likely present in form of reduced, stable Cr(III) complexes. The Cr isotope signatures in the rhodochrosite samples therefore still reflect the original water redox conditions.

In summary, the sediments of the Datangpo Formation in the Nanhua Basin record a stratified basin with a large anoxic bottom zone and an oxic near-surface reservoir, with turn-around events when pervasive weakly oxic episodes allowed manganese carbonate precipitation at the seafloor. The low amounts of redox-sensitive elements in both the manganese ore and the black shales suggest a low level of atmospheric oxygenation, but with periods of weak oxic conditions during the formation of manganese ore. Recent work on South China and elsewhere (ref.^46^) suggests fluctuating oxygenation states both on land and in the oceans after major glaciations. Our data build upon emerging evidence for the coupled links between bio-productivity levels, deposition of Mn and Fe-rich chemical sediments, and the oxygenation of Earth’s atmosphere during the Cryogenian interglacial period.

## Methods

### Cr isotope analysis

In this study we used drill core samples, which were powdered using an agate disk mill to minimize metal contamination from the milling process. For black shale bulk digests approximately 10 mg of sample was required, while the bulk rhodochrosite digests required 20 mg to 50 mg of sample (amounts adjusted to yield 1 µg Cr in the final separate). The powdered samples where weighed into ceramic crucibles and placed in the oven at 750 °C for 5 h to disintegrate the organic component of the samples. Subsequently the samples were transferred into pre-cleaned Teflon beakers and attacked by a concentrated HF-aqua regia mix on a hotplate at 125 °C overnight. In addition, leachates were performed for the rhodochrosite samples to minimize the effect of detrital dilution. For the leaches, between 200 mg and 300 mg of the powdered sample was reacted with 0.5 N HCl for ~2 h on a shaker table (amounts adjusted to yield 0.5 µg Cr in the final aliquot). Subsequently, the samples consisting of the centrifuged liquid phase were pipetted into pre-cleaned Teflon beaker and dried down on a hot plate at 130 °C. Finally, the dried down residues were re-dissolved in 6 N HCl, aliquoted and dried down again.

A ^50^Cr-^54^Cr double spike was added to the bulk and leachate samples in a sample to spike ratio of 4:1. The bulk samples were spiked after the oven and before the addition of the HF-aqua regia mix, while the leachates where spiked after aliquoting. To ensure spike-sample homogenization, the sample-spike mixtures were re-dissolved in aqua regia and dried down again. For bulk samples, chromium was separated in two chromatographic column steps, consisting of an anion and a cation column separation. The leachates on the other hand required the addition of another chromatographic column step, designed to remove iron before the anion and cation separations For this procedure, samples were re-dissolved in 1 ml 6 M HCl and passed over an anion exchange columns (BioRad) loaded with pre-cleaned Dowex AG 1 × 8 anion resin (100–200 mesh, pre-conditioned with 6 M HCl). Another 5 ml of 6 N HCl were added and the 6 N HCl eluates were collected and dried down on a hot plate at 130 °C. For the anion chromatographic column step, the samples were re-dissolved in 20 ml 0.1 N HCl, which were doped with 0.5 ml freshly mixed 1 M ammonium persulfate ((NH_4_)_2_S_2_O_8_) solution as an oxidizing agent. To ensure full oxidation of Cr(III) to Cr(IV), the sample solutions were placed on a hot plate at 130 °C for 1 h. The cooled samples were then passed over anion exchange columns (BioRad) loaded with pre-cleaned Dowex AG 1 × 8 anion resin (100–200 mesh, pre-conditioned with 0.1 M HCl). To wash-out the matrix, first 10 ml of 0.2 N HCl, then 2 ml of 2 N HCl and finally 5 ml of pure H_2_O were passed over the columns before Cr was collected through reduction with 6 ml 2 N HNO_3_ doped with a few drops of 5% H_2_O_2_. The collected Cr bearing eluates were then dried down at 130 °C overnight. For the cation chromatographic column step the Cr-bearing samples produced during the anion exchange chromatographic procedure were re-dissolved in 2.4 ml 0.5 M HCl. This solution was passed over cation columns loaded with 2 ml of pre-cleaned Dowex AG50W-X8 cation resin (200–400 mesh, pre-conditioned with 0.5 M HCl). Cr was released with 8 ml of 0.5 M HCl and Cr bearing solutions were dried down on a hot plate at 130 °C, before they could be loaded for Cr isotopic analysis on the thermal ionization mass spectrometer.

The Cr isotope measurements were carried out at the University of Copenhagen on a IsotopX/GV IsoProbe T or Phoenix thermal ionization mass spectrometer (TIMS) equipped with eight Faraday collectors. These were used for the simultaneous collection of the four chromium beams (^50^Cr^+^, ^52^Cr^+^, ^53^Cr^+^, ^54^Cr^+^) as well as the ^49^Ti^+^, ^51^V^+^ and ^56^Fe^+^ masses as monitors for the interferences on respective Cr masses. Chromium separates were loaded onto outgassed Re-filaments using a mix of 1 μl of 0.5 N H_3_PO_4_, 2.5 μl silicic acid and 0.5 μl of 0.5 N H_3_BO_3_ as a loading solution. Filament temperatures varied between 1050–1250 °C and the ^52^Cr beam intensities were kept at either 0.5 or 1 V. Two runs consisting of 120 cycles were run for every sample. The δ^53^Cr isotopic compositions of the samples were determined as the average of the repeated analysis and are reported in per mil (‰) ± double standard deviation (2σ) relative to the international standard reference material NIST SRM 979, reported as δ^53^Cr (‰) = ((^53^Cr/^52^Cr_sample_)/(^53^Cr/^52^Cr_NIST SRM 979_) − 1) × 1000. In cases where the 2σ errors were below the external 2σ reproducibility of double spiked NIST SRM 979 of 0.09‰, we employed and assigned that external reproducibility error to the respective analyses.

### Major-, trace elements, TOC and sulfur concentrations

The chemical dissolution of major elements was carried out in an ultraclean laboratory and measured using a Finnigan MAT ELEMENT high-resolution ICP-MS instrument. Approximately 100 mg of ~200 mesh powdered sample was digested with 1 ml HF and 0.5 ml HNO_3_ at 190 °C for 12 h. The solution was then drained and evaporated to dryness. The final residue was re-dissolved using 8 ml 40 vol.% HNO_3_. Subsequently, the sample was heated in an electric oven at 110 °C for 3 h. After cooling, the final solution was diluted with 100 ml distilled de-ionized water. The total analytical errors of the major element analyses are within ± 6% (1σ).

To measure the concentrations of trace elements, 25 mg powdered sample was digested in a sealed beaker using 1 ml 23 N HF and 0.5 ml 16 N HNO_3_ mix, and heated in the oven at 185°C overnight. After cooling down to room temperature, add 0.5 ml 16 N HNO_3_ into the beaker and dried the solution on the hotplate. This procedure is repeated twice. Subsequently, add 5 ml 50% HNO_3_ and sealed the beaker again, and heated at 130°C on a hotplate for 3 h. After cooling down, transform sample solution to a pre-cleaned beaker, and diluted the sample with 25 ml MQ-water for ICPMS measurement. The uncertainties for the trace element contents are between 5% and 10%.

The TOC contents were determined by potassium dichromate (K_2_Cr_2_O_7_) solution digestion technique. A total of 0.1 g powdered sample was mixed with 0.1 g silver sulfate (Ag_2_SO_4_) and added to 10 ml of a 0.4 mol/l K_2_Cr_2_O_7_-H_2_SO_4_ mixed solution. The sample was reacted at 175 °C for 5 minutes and transfer to a new beaker using 60–70 ml pure water. Next 5 ml of a 1.69 g/L H_3_PO_4_ solution was added into the beaker. Finally 0.2 mol/l FeSO_4_ solution was added to the solution until a neutral pH was achieved. The TOC content was then calculated from the required volume of the FeSO_4_ solution. The detection limit for TOC in this study is 0.02% with an uncertainty of <0.1%. The total sulfur (TS) contents were determined by a Leco infrared carbon/sulfur analyzer.

## Supplementary information

SUPPLEMENTARY INFORMATION

Supplementary table 1

Supplementary table 2

Supplementary table 3

Supplementary table 4
