# Supplementary material for: Subtle Cr isotope signals track the variably anoxic Cryogenian interglacial period with voluminous manganese accumulation and decrease in biodiversity
Source: Sci Rep. 2019 Oct 21;9:15056. doi: 10.1038/s41598-019-51495-0 (PMC6803686; doi:10.1038/s41598-019-51495-0)
Supplement: Supplementary file 1 — SUPPLEMENTARY INFORMATION [file 41598_2019_51495_MOESM1_ESM.pdf]

SUPPLEMENTARY INFORMATION

Subtle Cr isotope signals track the variably anoxic Cryogenian interglacial period with voluminous manganese accumulation and decrease in biodiversity

Lingang Xu[1], Anja B. Frank[2], Bernd Lehmann[3], Jianming Zhu[1], Jingwen Mao[1], Yongze Ju[4], Robert Frei[2]

[1]State Key Laboratory of Geological Processes and Mineral Resources, China University of Geoscience, 100083 Beijing, China
[2]Department of Geosciences and Natural Resource Management, University of Copenhagen, 1350 Copenhagen, Denmark
[3]Mineral Resources Unit, Technical University of Clausthal, 38678 Clausthal-Zellerfeld, Germany
[4]China Aero Geophysical Survey & Remote Sensing Center for Land and Resources, 100083 Beijing, China.

Supplementary figures

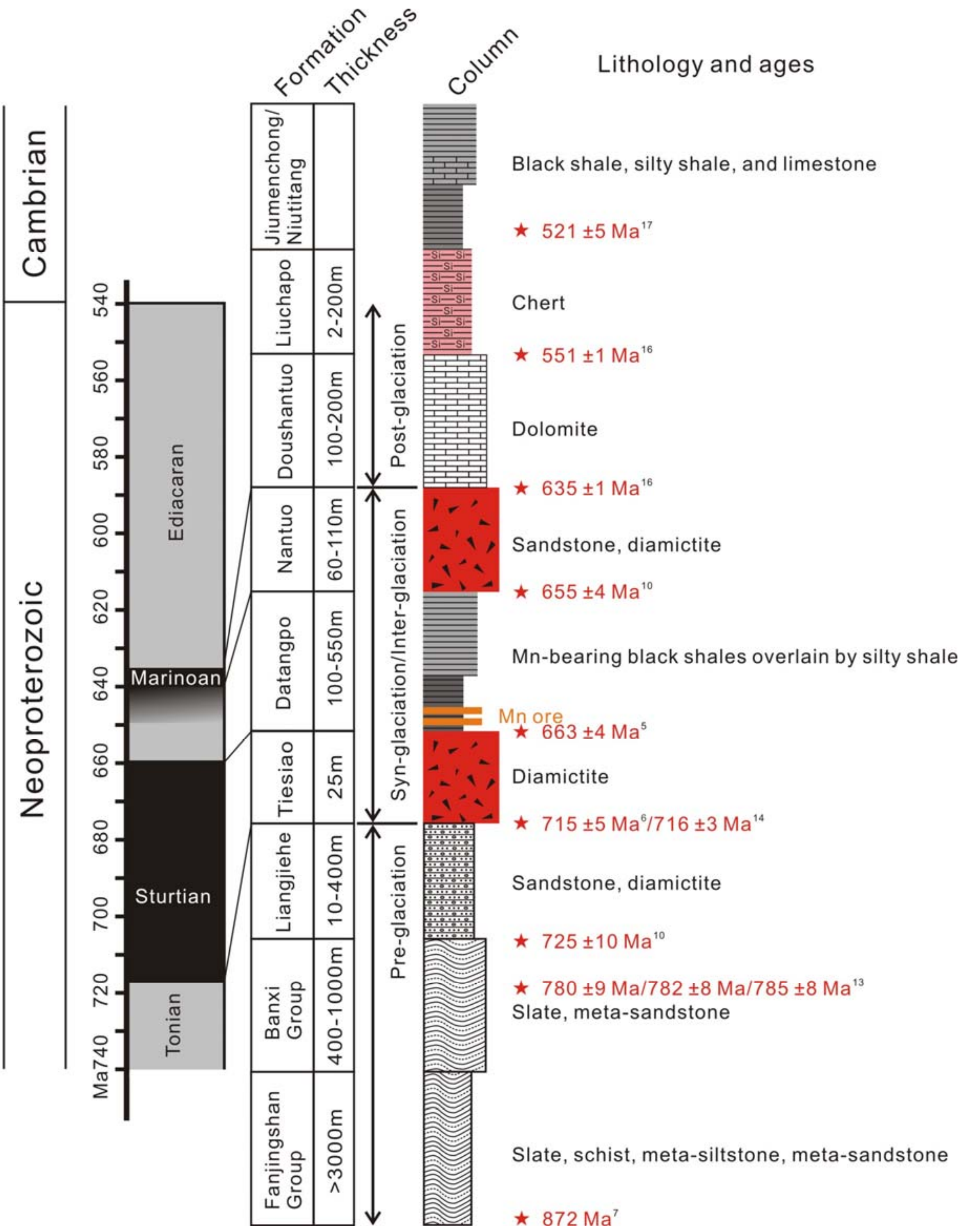

Supplementary Figure 1: Sketched stratigraphic column with radiometric age constraints in northeast Guizhou, Nanhua Basin, at the southeastern margin of the Yangtze Platform. The Tiesi`ao and Nantuo Formation are globally correlated with the Sturtian and Marinoan glaciation, respectively. The Datangpo Formation represents the Cryogenian interglacial interval that was bracketed by zircon U-Pb ages of 663 ±4 Ma and 655 ±4 Ma.

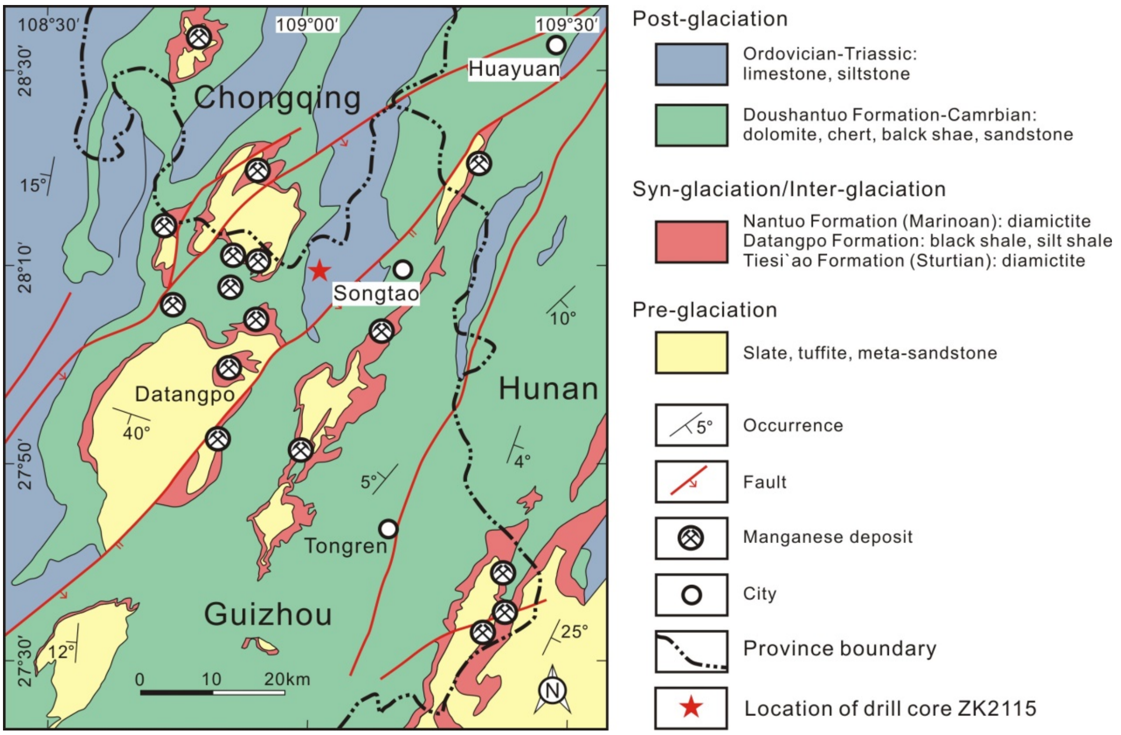

Supplementary Figure 2: Geological map of the manganese deposit cluster in the Nanhua Basin, South China, including the location of the drill core sampling. The Mn deposit cluster is located in the area where the Guizhou, Hunan, and Chongqing province intersect.

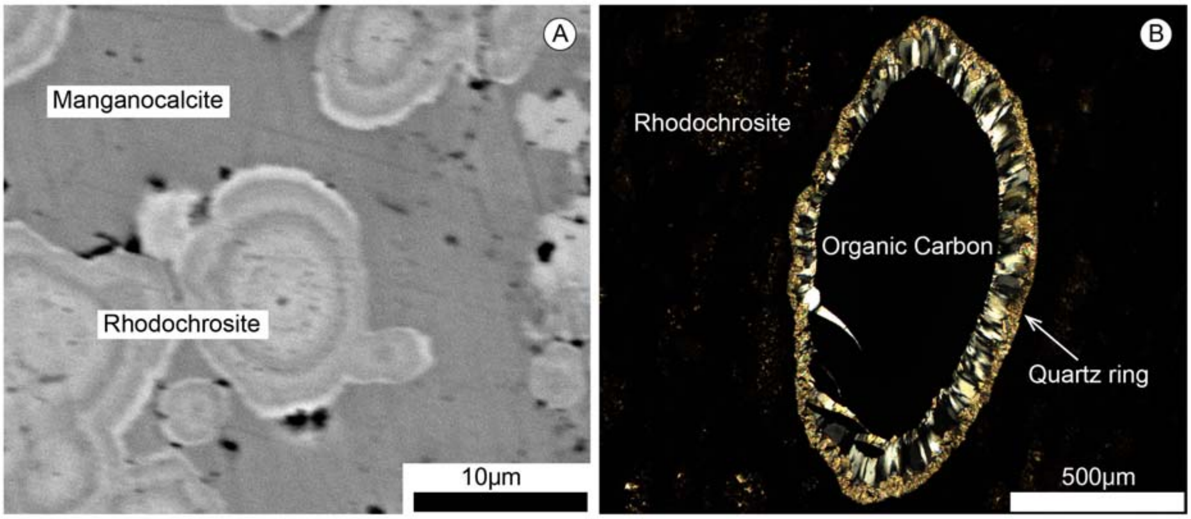

Supplementary Figure 3: (A): Oolitic texture of rhodochrosite cemented by manganocalcite matrix (dark color). The bright color rings surrounding the oolites denote high Mn components (reflected light). (B): Bubble-shaped texture in high grade massive Mn ores (transmitted cross-polar light). The core of the bubble is filled by organic carbon with comb-textured quartz rim.

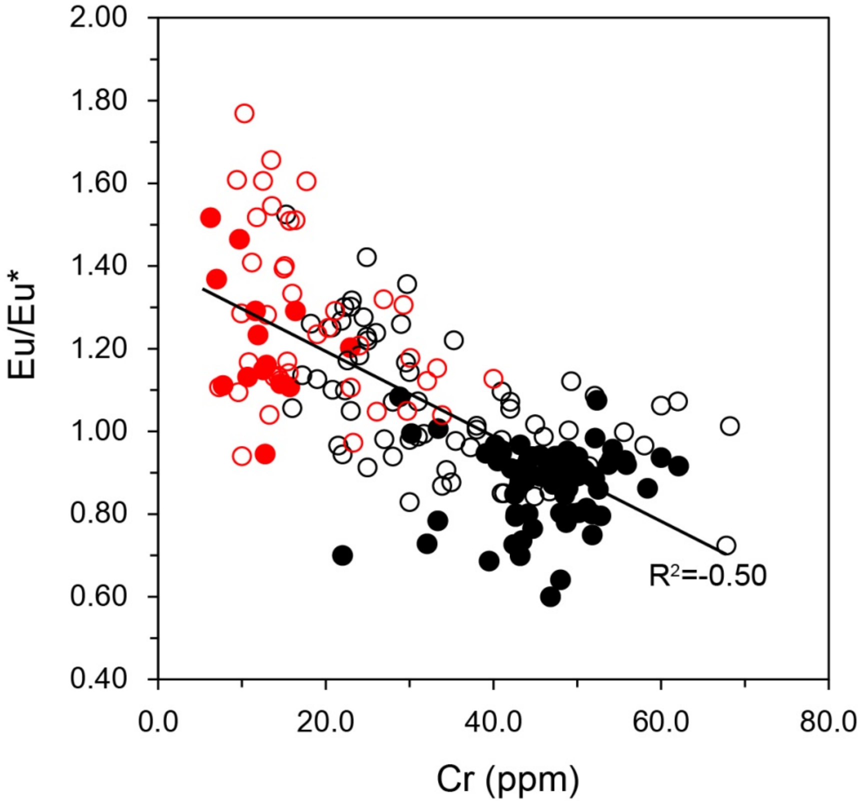

Supplementary Figure 4: The negative correlation between Cr contents and Eu/Eu* indicates that Cr was derived from the continentals by weathering. The solid red and black dots denote Mn ore and black shale samples, respectively, from this study. The open red and black circles represent data of Mn ore and black shale samples, respectively, from ref. [19] and [20].

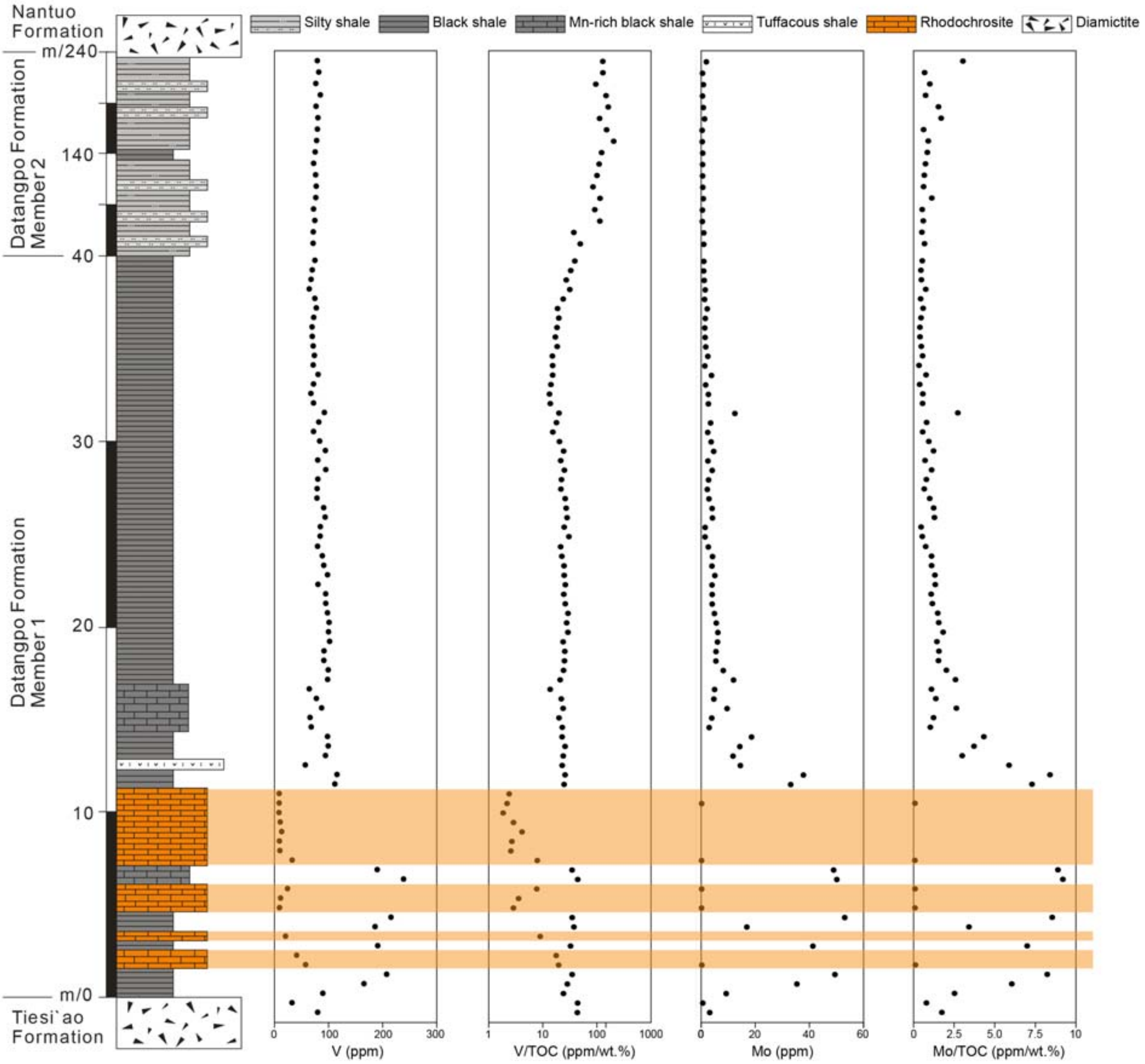

Supplementary Figure 5: Geochemical profiles of the interglacial Datangpo Formation from the Nanhua Basin, South China (Data are included in Supplementary table 1). The redox sensitive elements (Mo and V) and their ratios with TOC of black shales are consistently low until Ediacaran (Figure 1), with weak enrichment and elevation at the base of the Datangpo Formation, indicating generally low but fluctuating oxygenation conditions during the Sturtian-Marinoan interglacial interval. The light orange horizontal bars denote manganese ore layers.

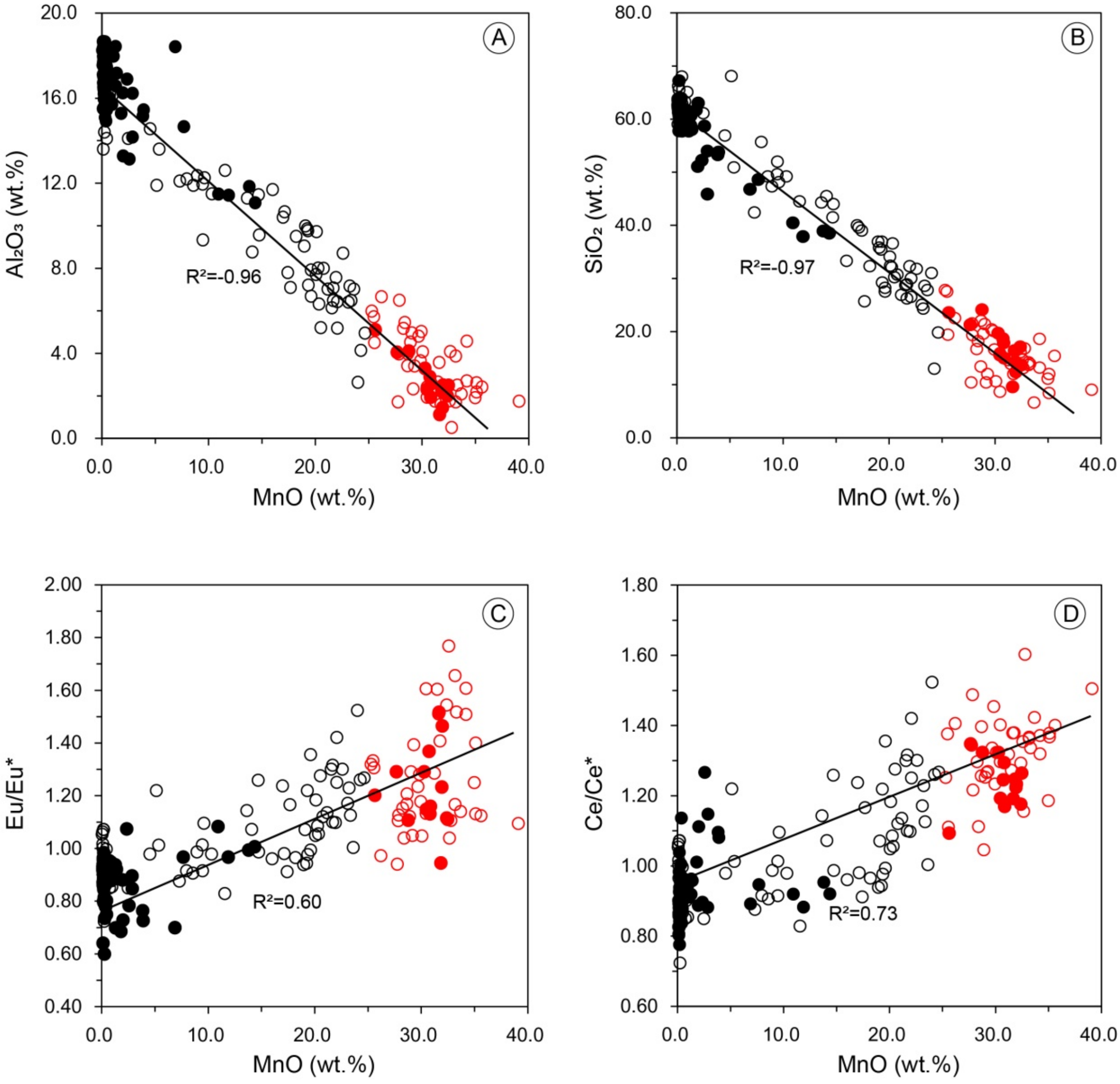

Supplementary Figure 6: Negative correlations between MnO and Al_2O_3 (A), and between MnO and SiO_2 (B), which indicate manganese ore samples are mixtures of detrital and authigenic components. Positive correlations between MnO and Eu/Eu* (C), and between MnO and Ce/Ce* (D), which suggest that the manganese carbonates precipitated by mixing of glacial-fed river water with seawater under weekly oxidized condition. The data of manganese ore samples and black shales from this study are represented by solid red and black dots, respectively. The data of manganese ore samples and black shales cited from ref. [19,20] are represented by open red and black circles, respectively.

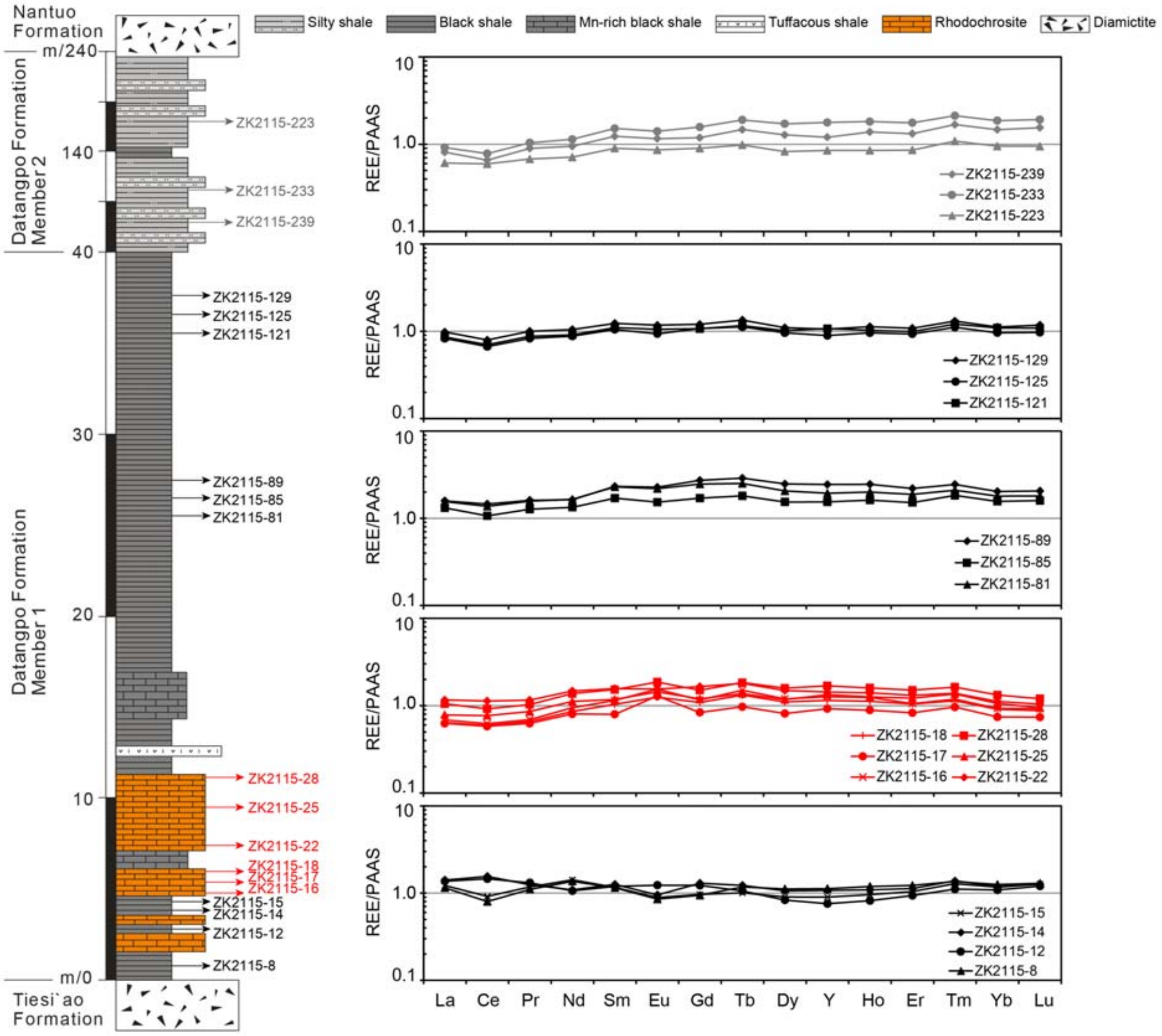

Supplementary Figure 7: PAAS-normalized REE patterns of representative manganese carbonates and black shales illustrated according to stratigraphic position. Black shales from Member 1 and silty shale from Member 2 are exhibited by black and gray symbols. Manganese ores are presented by red symbols. Positive Eu/Eu* anomalies and enrichment of middle REE indicate the model of mixing of glacial-fed river water with seawater.

Supplementary Note 1: Geological background

The South China Block formed by amalgamation of the Yangtze and Cathaysia blocks during the Sibao orogeny at ca. 1.1 -0.9 Ga[1,2]. The Nanhua Basin in South China represents an intracontinental rift basin that developed on the southeast-facing passive continental margin during Middle to late Neoproterozoic as a result of the breakup of Rodinia Supercontinent[3]. The post-rift extension of the South China Block created significant accommodation space in which thick Cryogenian successions were deposited in the Nanhua Basin[4]. In recent years, a large exploration programs in the Guizhou-Hunan-Chongqing province and adjacent areas focused on drilling of Mn-bearing rocks enabling the study of the sedimentation history of the Nanhua Basin. Detailed lithological correlation within the Nanhua Basin has been described by (ref. [4-6]) and references therein. In this study focusing on the Cryogenian interval, we divided the Late Neoproterozoic lithological units in the Nanhua Basin into three groups: pre-glaciation, syn-glaciation/inter-glaciation, and post-glaciation. In ascending order, the pre-glaciation unit (872 - 715 Ma) consists of the Fanjingshan Group, the Banxi Group, and the Liangjiehe Formation[6-8]; the syn-glaciation/inter-glaciation unit (715 - 635 Ma) consists of the Tiesi`ao Formation, the Datangpo Formation, and the Nantuo Formation; the post-glaciation unit (635 -541 Ma) consists of the Doushantuo Formation and the Liuchapo Formation, which is overlain by Phanerozoic rocks. Sediments deposited during the pre-glaciation stage consist mainly of more than 5000 m-thick greenschist-facies metamorphosed sandstones, siltstones, and shales with basalts and pillow lavas interlayers in the Fanjingshan Group and with tuff layers in the Banxi Group. The Banxi Group is unconformably overlain by the Fanjiangshan Group as a result of Jinning-Sibao tectono-orogenic movements in South China[9]. The Liangjiehe Formation is unconformably overlain by the Banxi Group and consists of gravel-bearing sandstones with dolomite interlayers. The syn-glaciation/inter-glaciation unit records continuously integrated marine deposition from the onset of the Sturtian glaciation to the terminal of the Marinoan glaciation. The Tiesi`ao and Nantuo Formation are globally corresponding to the Sturtian and Marinoan glaciation, respectively[10-12]. The 20 – 400 m thick Tiesi`ao Formation consists of poorly sorted diamictites with 10-15% in volume of mm- to cm-sized, low-degree-rounded pebbles. Approximately <1 m thick dolomicrite interlayers occur in the lower part of the Tiesi`ao Formation. The Nantuo Formation represents a fast precipitation of thick diamictites (approximately 300 m thick). Rounded and subangular pebble content is about 1-5 % in volume. The Datangpo Formation in between the Tiesi`ao and Nantuo Formation documents inter-glaciation sedimentation following the Sturtian glaciation in Nanhua Basin. It is subdivided into two lithological members. Member 1 (Mb.1) in the lower Datangpo Formation is meters to tens of meters thick, and consists of black shales with bedded rhodochrosite ore at the base. Approximately 80 – 350 m-thick Member 2 (Mb.2), consisting of siltstones, form as gradual change from Mb.1, with characteristically decreasing contents of TOC from Mb.1 to Mb.2. The cap carbonates of the Doushantuo Formation (20 - 60 m in thickness) documents the onset of post-glaciation sedimentation, overlain by Ediacarian cherts of the Liuchapo Formation.

Supplementary Note 2: Geochronological framework

In recent years, a high-precision chronological framework has been established for the Late

Neoproterozoic strata exposed in the Nanhua Basin, South China (Supplementary Fig. 1). On the base of detrital zircon U-Pb dating using the LA-ICPMS technique, the sedimentation of the Fanjingshan Group is suggested to start ~ 872 Ma[7]. Zircon SHRIMP U-Pb ages of 785 ±8 Ma, 782 ±8 Ma, and 780 ±9 Ma have been reported for tuff layers from the middle Banxi Group[13], in line with a zircon SHRIMP U-Pb age of 725 ±10 Ma for a tuff bed at the top of the Banxi Group[10]. The onset of the Tiesi`ao/Changan glaciation (Sturtian glaciation equivalent) in the Nanhua Basin has been constrained to 716 ±3 Ma using SIMS dating on zircons from a tuffaceous siltstone bed below the diamictite unit[14]. This age is in good agreement with a newly reported youngest detrital zircon LA-ICPMS age of 715 ±5 Ma for the sandstone that underlies the Tiesi`ao/Changan glacigenic sediments[6], indicating that the Sturtian glaciation in the Nanhua Basin started from ~715 Myr ago. Zhou et al. (2004) (ref. [5]) recognized tuff beds at the lower part of the Datangpo Formation in the manganese ore field of the Guizhou province, and a zircon U-Pb age of 663 ±4 Ma (using TIMS) was reported from this unit. This age denotes the terminal age of Sturtian glaciation in South China. A SHRIMP U-Pb zircon age of 655 ±4 Ma from an ash bed immediately below the Nantuo glaciation (Marinoan glaciation equivalent) provides the upper age constraint for the inter-glaciation unit represented by the Datangpo Formation[10]. Recently, cyclostratigraphic studies also suggests fast (<10 Myr) sedimentation of the Datangpo Formation[15]. U-Pb zircon dates from volcanic ash beds within the Doushantuo Formation indicate that the deposition occurred between 635 ±1 Ma and 551 ±1 Ma[16]. The Liuchapo Formation overlying the Doushantuo Formation overlaps with the Neoproterozoic - Phanerozoic boundary. An organic-, sulfide-rich layer above the Neoproterozoic Cambrian boundary has been dated at 521 ±5 Ma using the Re-Os dating method, and serves as a marker layer for regional comparisons[17].

Supplementary Note 3: Ore deposit geology

The Guizhou-Hunan-Chongqing province and adjacent areas have been approved as an economically significant Mn ore field in South China, where several giant Mn ore deposits have been recently discovered (Supplementary Fig. 2). Total Mn reserves from this area have been calculated at over one billion tons and these will increase with increasing exploration work[18]. Manganese ores are all hosted in black shales of the lower part of the Datangpo Formation, in Mb.1, as stratiform ore bodies a few centimeters above the Tiesi`ao diamictites. Taking MnO_2 contents over 25% as the threshold for a manganese ore, several layers of such ore can be identified. Interlayers are either manganiferous shale or barren black shales. Total thicknesses of Mn ore layers are variable, but a thick ore body in the center of the basin (up to 14 m thick) is usually followed by decreasingly thick layers towards the basin margins[18]. Rhodochrosite is the most dominate ore mineral, with manganocalcite occurring as matrix. Gangue minerals are mainly quartz, feldspar, dolomite, illite, and minor apatite and bastnaesite[19]. Pyrite is common both in the manganese ores and black shales. It occurs as euhedral-subhedral and framboidal grains and aggregates. A tuffaceous sandstone layer with variable thickness (centimeters to 3 meters) occurs within the Mn ore layers. Both lenticular and laminated ore are found in the Mn ore beds. Rhodochrosite displays oolitic texture consisting of ring aggregates with high Mn concentration and manganocalcite as cement (Supplementary Fig 3A). In the massive, high Mn grade ore, “bubble shaped” textures are common and consist of carbonaceous nodules enveloped by comb-shape quartz (Supplementary Fig. 3B).

Supplementary Note 4: Sampling

Samples analyzed in this study were collected from a drill core (ZK2115) from within the northeast Guizhou Province, performed as a Mn ore exploration drill. The drill site coordinates are 28°07′30″N and 108°51′34″E. We took samples avoiding visible pyrite or quartz veins, and excluded pieces with fractures to circumvent potential contamination by later hydrothermal fluids. Our samples cover the interval between the diamictites of the upper part of Tiesi`ao Formation and the sediments of the upper Datangpo formation, thus covering the entire inter-glaciogneic sedimentation in the basin. The depth of each sample from the surface is indicated in Supplementary Excel File. In addition, two diamictite samples from the Tiesi`ao Formation were collected at 1648.0 m (ZK2115-1) and 1644.0 m (K2115-3) depth. The boundary between the diamictite of the Tiesi`ao Formation and black shale of the Datangpo Formation in the sampled drill core occurs at 1637.3 m. The stratigraphic sampling intervals were different, and they are ~ 0.5 m for Mb.1 (1636.5 – 1596.2 m), ~ 1.5 m for the lower part of Mb. 2 (1595.7 – 1590.0 m), and ~ 10.9 m for the upper part of Mb.2 (1579.1 – 1415.5 m). We took fresh bulk samples from the drill cores, in the order of 100 -200 g pieces, which then were crushed and powdered with an agate shatter box prior to chemical analysis.

Supplementary Note 5: Analytical data

The complete data sets of both major and trace element concentrations and Cr isotope compositions are listed in Supplementary Excel File. Rare earth element and yttrium (REE + Y) concentrations are normalized to Post-Archean Australian Shale (PAAS)[21]. To avoid any potential influence caused by La and Gd anomalies that are a characteristic feature of marine samples, we calculated Ce and Eu anomalies using the following equations[22]:

$Ce/Ce^* = Ce_n/(Pr_n^2 \times /Nd_n)$

and

$Eu/Eu^* = Eu_n/(Sm_n^2 \times Tb_n)^{1/3}$, where subscript "n" refers to PAAS normalized values.

5.1 Tiesi`ao Formation

Two diamictite samples (ZK2115-1 and -3) from the Tiesi`ao Formation have bulk $\delta^{53}Cr$ values of -0.20 ±0.09 ‰ and -0.05 ±0.09 ‰. They also contain 17.45 and 8.25 wt.% Al_2O_3, 4.35 and 3.91 wt.% Fe_2O_3, 0.05 and 1.09 wt.% MnO, 0.27 and 0.20 wt.% P_2O_5, 1.80 and 0.69 wt.% TOC, and 2.52 and 0.80 wt.% TS. Selected trace element concentrations are of these two diamictites are: 39.0 and 16.7 ppm Cr, 79.4 and 30.8 ppm V, 3.04 and 0.50 ppm Mo, and 2.65 and 0.85 ppm U. Ce/Ce* values are 0.92 and 0.94, respectively. Eu/Eu* values are 0.80 and 1.03, respectively.

5.2 Mb.1 of the Datangpo Formation

On the basis of MnO content, we classify samples into manganese ore (MnO ≥ 25 wt.%) and manganiferous host black shale (MnO < 25 wt.%). The manganese ore is characterized by positively fractionated Cr isotope signatures with $\delta^{53}Cr$ values varying from -0.17 to 0.22 ‰

(0.01 ±0.11 ‰ on average) for bulk samples and from -0.07 to 0.24 ‰ (0.11 ±0.10 ‰ on average) for leachates. Major element characteristics of manganese ore are: 1) low Al_2O_3 contents (1.12 to 5.10 wt.%, 2.72 wt.% on average) and low Fe_2O_3 contents (1.81 to 4.41 wt.%, 2.36 wt.% on average); 2) high MnO contents (25.64 to 32.49 wt.%, 30.54 wt.% on average) and relatively high P_2O_5 contents (0.21 to 0.88 wt.%, 0.34 wt.% on average). TOC and TS contents of manganese ores vary from 2.09 to 3.94 wt.% (2.86 wt.% on average) and from 0.49 to 2.57 wt.% (0.93 wt.% on average), respectively. Trace metal concentrations (including redox-sensitive elements) are relatively low: 6.27 to 22.9 ppm Cr (12.4 ppm on average), 6.37 to 56.7 ppm V (17.1 ppm on average), and less than 0.13 ppm Mo. Both Ce and Eu show prominent positive anomalies with Ce/Ce* and Eu/Eu* values varying from 1.09 to 1.35 (1.24 on average) and 0.94 to 1.52 (1.22 on average), respectively. La_n/Nd_n and Gd_n/Yb_n ratios of manganese ores vary from 0.67 to 0.79 (0.73 on average) and from 0.98 to 1.48 (1.15 on average), respectively, exhibiting weak enrichment of middle REE.

Compared with the manganese ore, the black shales show unfractionated Cr isotope compositions similar to bulk silicate earth values, with $\delta^{53}Cr$ values varying from -0.20 to 0 ‰ (-0.09 ±0.05 on average) in bulk samples. Two Mn-rich black shales (ZK2115-41 and -47), leached in the same way as the rhodochrosite ore samples, also revealed unfractionated Cr isotope compositions in their leachates, with $\delta^{53}Cr$ value of -0.12 ±0.09‰ and -0.14 ±0.09‰, respectively. Relative to the manganese ores, the black shales have: 1) high Al_2O_3 contents (11.1 to 18.7 wt.%, 16.3 wt.% on average) and high Fe_2O_3 contents (3.25 to 8.17 wt.%, 5.52 wt.% on average); 2) low MnO contents (0.13 to 14.4 wt.%, 1.73 wt.% on average) and low P_2O_5 contents (0.05 to 1.03 wt.%, 0.18 wt.% on average), and 3) high TOC contents (1.88 to 6.17 wt.%, 3.97 wt.% on average) and high TS contents (1.49 to 6.08 wt.%, 2.93 wt.% on average). The black shales contain relatively high trace metal concentrations: 22.0 to 99.1 ppm Cr (46.1 ppm on average), 56.0 to 242 ppm V (96.4 ppm on average), and 0.81 to 52.7 ppm Mo (9.58 ppm on average). In contrast to the manganese ore samples, black shales lack and/or only have weak positive Ce anomalies, with Ce/Ce* varying from 0.83 to 1.27 (0.93 on average). Black shales with weak positive Ce anomalies are mostly from manganese ore interlayers. In addition, the black shales display weak negative Eu anomalies with Eu/Eu* ratios varying from 0.60 to 1.08 (0.87 on average), compatible with average continental crust derivate. La_n/Nd_n and Gd_n/Yb_n ratios of black shales vary from 0.84 to 1.29 (0.99 on average) and from 0.75 to 1.52 (1.10 on average), respectively.

5.3 Mb.2 of Datangpo Formation

Similar to the black shales from the Mb.1, silty shale samples from the Mb.2 of the Datangpo Formation also have consistently unfractionated Cr isotope signatures, with $\delta^{53}Cr$ values varying from -0.12 to -0.07 ‰ (-0.10‰ on average). The silty shales are characterized by the following major element oxide concentrations: 15.4 to 18.7 wt.% Al_2O_3 (17.8 wt.% on average), 4.43 to 10.2 wt.% Fe_2O_3 (6.42 wt.% on average), 0.12 to 0.42 wt.% MnO (0.21 wt.% on average), and 0.05 to 0.13 wt.% P_2O_5 (0.10 wt.% on average). TOC and TS contents of the silty shales range from 0.37 to 3.17 wt.% (0.94 wt.% on average) and from 0.05 to 3.95 wt.% (0.76 wt.% on average), respectively, and are lower than that those of the black shales from Mb.1. The silty shales contain 43.2 to 62.1 ppm Cr (51.1 ppm on average), 60.9 to 84.5 ppm V (73.8

ppm on average), and 0.28 to 1.80 ppm Mo (0.64 ppm on average). Weak negative Ce and Eu anomalies are characteristic of the silty shales, with Ce/Ce* and Eu/Eu* varying from 0.78 to 1.05 (0.90 on average) and from 0.64 to 1.25 (0.91 on average), respectively. La_n/Nd_n and Gd_n/Yb_n ratios of silty shales vary from 0.75 to 1.57 (0.99 on average) and from 0.71 to 1.40 (0.97 on average), respectively.

References:

1. Li, Z.X., Li, X.H., Zhou, H.W. & Kinny, P.D. Grenvillian continental collision in south China: new SHRIMP U-Pb zircon results and implications for the configuration of Rodinia. Geology **30**, 163-166 (2002).
2. Ye, M.F., Li, X.H., Li, W.X., Liu, Y. & Li, Z.X. SHRIMP zircon U-Pb geochronological and whole-rock geochemical evidence for an early Neoproterozoic Sibaoan magmatic arc along the southeastern margin of the Yangtze Block. Gondwana Research **12,** 144-156 (2007).
3. Wang, H., Chu, X., Liu, B., Hou, H. & Ma, L., eds. Atlas of the paleogeography of China: Beijing, Cartographic Publishing House, 143 (1985).
4. Wang, J. & Li, Z.X. History of Neoproterozoic rift basins in South China: implications for Rodinia break-up. Precambrian Research **122,** 141-158 (2003).
5. Zhou, C., Tucker, R., Xiao, S., Peng, Z., Yuan, X. & Chen, Z. New constraints on the ages of Neoproterozoic glaciations in south China. Geology **32,** 437-440 (2004).
6. Song, G., Wang, X., Shi, X. & Jiang, G. New U-Pb age constraints on the upper Banxi Group and synchrony of the Sturtian glaciation in South China. Geoscience Frontiers **8,** 1161-1173 (2017).
7. Zhou, J.-C., Wang, X.-L. & Qiu, J.-S. Geochronology of Neoproterozoic mafic rocks and sandstones from northeastern Guizhou, South China: Coeval arc magmatism and sedimentation. Precambrian Research **170,** 27-42 (2009).
8. Lan, Z., Li, X.-H., Zhu, M., Zhang, Q. & Li, Q.-L. Revisiting the Liantuo Formation in Yangtze Block, South China: SIMS U-Pb zircon age constrains and regional global significance. Precambrian Research **263,** 123-141 (2015).
9. Zhao, G. & Cawood, P.A. Precambrian geology of China. Precambrian Research **222-223,** 13-54 (2012).
10. Zhang, Q.-R., Li, X.-H., Feng, L.-J., Huang, J. & Song, B. A new age constraint on the onset of the Neoproterozoic glaciations in the Yangtze Platform, South China. Journal of Geology **116,** 423-429 (2008).
11. Macdonald, F.A., Schmitz, M.D., Crowley, J.L., Roots, C.F., Jones, D.S., Maloof, A.C., Strauss, J.V., Cohen, P.A., Johnston, D.T. & Schrag, D.P. Calibrating the cryogenian. Science **327,** 1241-1243 (2010).
12. Zhang, F., Zhu, X., Yan, B., Kendall, B., Peng, X., Li, J., Algeo, T.J. & Romaniello, S. Oxygenation of a Cryogenian ocean (Nanhua Basin, South China) revealed by pyrite Fe isotope compositions. Earth and Planetary Science Letters **429,** 11-19 (2015).
13. Wang, Z., Wang, J., Duan, T., Xie, Y., Zhuo, J. & Yang, P. Geochronology of middle Neoproterozoic volcanic deposits in Yangtze Craton interior of South China and its implications to tectonic settings. Science China Earth Sciences **53,** 1307-1315 (2010).
14. Lan, Z., Li, X., Zhu, M., Chen, Z., Zhang, Q., Li, Q., Lu, D., Liu, Y. & Tang, G. A rapid and

synchronous initiation of the wide spread Cryogenian glaciations. Precambrian Research **255,** 401-411 (2014).

15. Bao, X., Zhang, S., Jiang, G., Wu, H., Li, H., Wang, X., An, Z. & Yang, T. Cyclostratigraphic constraints on the duration of the Datangpo Formation and the onset age of the Nantuo (Marinoan) glaciation in South China. Earth and Planetary Science Letters **483,** 52-63 (2018).
16. Condon, D., Zhu, M.Y., Bowring, S., Wang, W., Yang, A.H. & Jin, Y.G. U-Pb ages from the Neoproterozoic Doushantuo Formation, China. Science **308,** 95-98 (2005).
17. Xu, L., Lehmann, B., Mao, J., Qu, W. & Du, A. Re-Os age of polymetallic Ni-Mo-PGE-Au mineralization in Early Cambrian black shales of South China – a reassessment. Economic Geology **106,** 511-522 (2011).
18. Zhou, Q., Du, Y., Yuan, L., Zhang, S., An, Z., Pan, W., Yang, B., Xie, X., Yu, W., Yin, S., Wang, P., LV, D. & Xu, Y. Major progress and potential prediction of geological exploration in Songtao manganese National Fully Equipped Exploration District in Tongren, Guizhou. Guizhou Geology **33,** 237-244 (in Chinese) (2016).
19. Yu, W., Algeo, T.J., Du, Y., Maynard, B., Guo, H., Zhou, Q., Peng, T., Wang, P. & Yuan, L. Genesis of Cryogenian Datangpo manganese deposit: Hydrothermal influence and episodic post-glacial ventilation of Nanhua Basin, South China. Palaeogeography, Palaeoclimatology, Palaeoecology **459,** 321-337 (2016).
20. Wu, C., Zhang, Z., Xiao, J., Fu, Y., Shao, S., Zheng, C., Yao, J. & Xiao, C. Nanhuan manganese deposits within restricted basins of the southeastern Yangtze Platform, China: Constraints from geological and geochemical evidence. Ore Geology Reviews **75,** 76-99.
21. McLennan, S.M. Rare earth elements in sedimentary rocks: Influence of provenance and sedimentary processes. Reviews in Mineralogy and Geochemistry **21,** 169-200 (2016).
22. Lawrence, M.G., Greig, A., Collerson, K.L. & Kamber, B.S. Rare earth element and yttrium variability in south east Queensland waterways. Aquatic Geochemistry **12,** 39-72 (2006).
23. Cole, D.B., Reinhard, C.T., Wang, X., Gueguen, B., Halverson, G.P., Gibson, T., Hodgskiss, M.S.W., McKenzie, N.R., Lyons, T.W. & Planavsky, N.J. A shale-hosted Cr isotope record of low atmospheric oxygen during the Proterozoic. Geology **44,** 555-558 (2016).
24. Planavsky, N.J., Reinhard, C.T., Wang, X., Thomson, D., McGoldrick, P., Rainbird, R.H., Johnson, T., Fischer, W.W. & Lyons, T.W. Low Mid-Proterozoic atmospheric oxygen levels and the delayed rise of animals. Science **346,** 635-638 (2014).
25. Frei, R., Gaucher, C., Poulton, S.W. & Canfield, D.E. Fluctuations in Precambrian atmospheric oxygenation recorded by chromium isotopes. Nature **461,** 250-254 (2009).
26. Wei, W., Frei, R., Klaebe, R., Li, D., Wei, G. & Ling, H. Redox condition in the Nanhua Basin during the waning of the Sturtian glaciation: A chromium-isotope perspective. Precambrian Research, https://doi.org/10.1016/j.precamres.2018.02.009 (2018).
27. Frei, R., Døssing, L.N., Gaucher, C., Boggiana, P.C., Frei, K.M., Árting, T.B., Crowe, S.A. & Freitas, B.T. Extensive oxidative weathering in the aftermath of a late Neoproterozoic glaciation – Evidence from trace element and chromium isotope records in the Urucum district (Jacadigo Group) and Puga iron formation (Mato Gross do Sul, Brazil). Gondwana Research **49,** 1-20 (2017).
28. Rodler, A.S., Frei, R., Gaucher, C. & Germs, G.J.B. Chromium isotope, REE and redox-sensitive trace element chemostratigraphiy across the late Neoproterozoic Ghaub glaciation, Otavi Group, Namibia. Precambrian Research **286,** 234-249 (2016).

29. Wei, W., Frei, R., Gilleaudeau, G.J., Li, D., Wei, G.-Y., Chen, X. & Ling, H.-F. Oxygenation variations in the atmosphere and shallow seawater of the Yangtze Platform during the Ediacaran Period: Clues from Cr-isotope and Ce-anomaly in carbonates. Precambrian Research **313,** 78-90 (2018).
30. Frei, R., Gaucher, C., Stolper, D. & Canfield, D.E. Fluctuations in late Neoproterozoic atmospheric oxidation – Cr isotope chemostratigraphy and iron speciation of the late Ediacaran lower Arroyo del Soldado Group (Uruguay). Gondwana Research **23,** 797-811 (2013).
31. Dahl, T.W., Hammarlund, E.U., Anbar, A.D., Bond, D.P.G., Gill, B.C., Gordon, G.W., Knoll, A.H., Nielsen, A.T., Schovsbo, N.H. & Canfield, D.E. Devonian rise in atmospheric oxygen correlated to the radiations of terrestrial plants and large predatory fish. Proceedings of the Natural Academy of Sciences **107,** 17911-17915 (2010).
32. Guo, Q., Shields, G.A., Liu, C., Strauss, H., Zhu, M., Pi, D., Goldberg, T. & Yang, X. Trace element chemostratigraphy of two Ediacaran-Cambrian successions in South China: Implications for organosedimentary metal enrichment and silicification in the early Camrbian. Palaeogeography, Palaeoclimatology, Palaeoecology **254,** 194-216 (2007).
33. Sahoo, S.K., Planavsky, N.J., Kendall, B., Wang, X., Shi, X., Scott, C., Anbar, A.D., Lyons, T.W. & Jiang, G. Ocean oxygenation in the wake of the Marinoan glaciation. Nature **489,** 546-549 (2017).
34. Paikaray, S., Banerjee, S. & Mukherji, S. Geochemistry of shales from the Paleoproterozoic to Neoproterozoic Vindhyan Supergroup: Implications on provenance, tectonics and paleoweathering. Journal of Asian Earth Sciences **32,** 34-48 (2008).
35. Laskowski, N. & Kröner, A. Geochemical characteristics of Archaean and Late Proterozoic to Palaeozoic fine-grained sediments from Southern Africa and significance for the evolution of the continental crust. Geologische Rundschau **74,** 1-9 (1985).
36. Wang, X., Jiang, G., Shi, X., Peng, Y. & Morales, D.C. Nitrogen isotope constrains on the early Ediacaran ocean redox structure. Geochimica et Cosmochimica Acta **240,** 220-235 (2018).
37. Jin, C., Li, C., Algeo, T.J., O`Connell, B., Cheng, M., Shi, W., Shen, J. & Planavsky, N.J. Highly heterogeneous "poikiloredox" conditions in the early Ediacaran Yangtze Sea. Precambrian Research **311,** 157-166 (2018).
38. Li, C., Love, G.D., Lyons, T.W., Scott, C.T., Feng, L., Huang, J., Chang, H., Zhang, Q. & Chu, X. Evidence for a redox stratified cryogenian marine basin, Datangpo Formation, South China. Earth and Planetary Science Letters **331-332**, 246-256 (2012).

Supplementary tables are in excels

Supplementary table 1: Complete Cr isotope and geochemical data of drill core ZK2115 from the Nanhua Basin.

Supplementary table 2: Compiled geochemical data of manganese ore and host black shales from the Nanhua Basin.

Supplementary table 3: Compilation of sediment $\delta^{53}Cr$ values and Cr/Ti ratios across the 750 – 540 Ma interval.

Supplementary table 4: Compilation of Mo, V, and TOC compositions of black shales across the 750-540 Ma interval.
